# National oral health survey on refugees in Germany 2016/2017: caries and subsequent complications

**DOI:** 10.1007/s00784-020-03563-3

**Published:** 2020-10-04

**Authors:** A. Al-Ani, M. Takriti, J. Schmoeckel, M. Alkilzy, C. H. Splieth

**Affiliations:** Department of Preventive and Pediatric Dentistry, University of Greifswald, Fleischmannstr. 42, 17475 Greifswald, Germany

**Keywords:** Oral health, Refugees, Germany, Caries, Decayed, Missing and filled teeth index, Resident

## Abstract

**Objectives:**

To assess oral health, caries prevalence, and subsequent complications among recently arrived refugees in Germany and to compare these findings with the German resident population.

**Methods:**

This multicenter cross-sectional study recruited 544 refugees aged 3–75+ years; they were examined at ten registration institutions in four federal states in Germany by two calibrated dentists. The refugees were screened for caries (dmft/DMFT) and its complications (pufa/PUFA); this data was compared to the resident population via the representative national oral health surveys).

**Results:**

The deciduous dentition of the 3-year-old refugees had a mean dmft value of 2.62 ± 3.6 compared with 0.48 dmft in the German resident population, and caries increased to 5.22 ± 3.4 for 6–7-year-olds (Germany: 1.73 dmft). Few refugee children had naturally healthy teeth (7% in 6–7-year-olds, Germany: 56%). In the permanent dentition, the gap in caries prevalence between refugees and the German population decreased with age (35–44-year-olds: 10.55 ± 7.1 DMFT; Germany: 11.2), but refugees exhibited more caries defects (35–44-year-olds DT = 3.13 ± 3.0; Germany: 0.5). German residents had more restorations (35–44-year-olds FT = 4.21 ± 4.6). Regarding complications, the 6–7-year-olds exhibited the highest pufa index (0.86 ± 1.4) which decreased in adolescence (13–17-year-olds, 0.18 ± 0.6) and increased in adults (45–64-year-olds, 0.45 ± 0.8).

**Conclusion:**

The refugees had high caries experience, often untreated caries teeth and more complications compared with the German resident population, especially in children. Closing this gap by extending preventive systems to the refugees would decrease future treatment needs.

**Clinical relevance:**

European countries should be prepared for the higher dental treatment needs in recent refugees, especially in children.

## Introduction

The United Nations High Commissioner for Refugees counts 25.4 million refugees in its statistics, with numbers rising significantly in recent years as a result of the wars in Syria, Afghanistan, Iraq, Ukraine, Yemen, and many other countries worldwide [1]. Trying to reach a safe country, Canada, Australia, USA, Lebanon, Turkey, and many other countries are their destinations. Recently, Europe and especially Germany were affected by refugees in 2015/2016 according to the International Organization for Migration [2]. It was estimated that far more than one million refugees have arrived in the country, provisionally counted in the “EASY” system before applying for asylum [3]. Most applications in 2016 came from Syria (44.0%), Afghanistan (15.6%), and Iraq (14.5 %).

The countries where most of the refugees come from are unstable and often suffer from limited access to general and oral health systems. According to the World Health Organization, those countries show high rates of poverty, bad oral hygiene, and caries [4]. The immense burden of those refugees leaving their homes and countries, facing countless unknown and often life-threatening situations, has a negative impact on their physical, mental, and emotional health [5]. This also affects dental health since the access to care was already limited in their countries, decreased even more during their journey, and they face considerable barriers in their host country such as language and socioeconomic or cultural beliefs [6].

During the process of application for asylum, refugees in Germany are entitled to acute pain treatment [7]; after recognition, they are insured in the complete, free of charge German National Health System. Thus, huge numbers of refugees often impose a burden on their host countries financially, demographically, and strategically [8]. Up until now, there is little, selected data on oral health in refugees and their dental needs, but this would be necessary for planning, managing, and estimating future needs and costs for oral health care.

Therefore, the aims of this study were to assess caries and its subsequent complications in 3–75-year-old refugees in Germany and to compare their oral morbidity with data from national surveys on the German resident population.

## Material and methods

This study was carried out as a representative, multicenter, cross-sectional study of oral health and treatment needs among refugees in Germany after an approval by the Ethics Committee of the University of Greifswald (Reg. No. BB 021/17), following the guidelines and recommendations for securing good epidemiological practice [9].

Two calibrated and Arabic- and English-speaking dentists interviewed and examined 544 participants between December 2016 and May 2017. At the time of recruiting process, there was at least one initial reception center in each federal state in Germany from where the refugees were distributed proportionally among the federal states after registration. For 2018, the BAMF website (BAMF: Bundesamt für Migration und Flüchtlinge/Federal Office for Migration and Refugees) listed 68 offices in 64 locations [3]. We tried to contact the maximum number of those centers, preferably with high numbers of asylum seekers and refugees. Often, our requests were rejected due to security reasons, the lack of resources, or time under the high burden of incoming refugees. Thus, we received 10 positive replies from central accommodation facilities in 4 different federal states in Germany. Those four federal states were Mecklenburg-Pomerania, North Rhine-Westphalia, Berlin, and Hessia. Due to the by law proportional distribution of refugees, a selection bias is very unlikely. After contacting the management of the refugees’ centers for finding a suitable date for the recruiting, flyers were placed on the advertisements board, and the staff informed all refugees about free dental check-ups and advice on oral health care and treatment. The examining dentists also went to the rooms of the refugees door to door and asked them to join in the study in order to get their dental health get checked up. Informed consent was explained for all individual participants included in the study, and a signature of their approval was obtained. There was a very good acceptance for this procedure, especially as the refugees also received a free toothbrush, toothpaste, and advice for oral hygiene. Young children also got a small toy, and their parents were instructed in performing tooth brushing on their children.

Since most of the refugees were men aged 18–34 years old, there was a planned over-recruiting in children, women, and older adults to increase the validity of the data for these groups. Still, only few 65–74-year-olds were found among the refugees (*n* = 5) making a subanalysis for this age group not feasible.

After recording their age, gender, and country of origin, the clinical oral examination was performed on a chair near the window, but not in direct sunlight, with disposable dental instruments (dental mirror, explorer) and an additional bright headlight.

The examiners were calibrated and followed [4] standards which were also used in the National German Oral Health Surveys for caries diagnostics with the dmft/DMFT index and its subcomponents [6, 10, 11]. The calibration was done prior to the study after instructions on the WHO-based diagnostic criteria as well as the other parameters of the last National German Oral Health Survey, and then a sample of 20 refugees was examined by both dentists individually comparing the reliability. There was good to very high inter-examiner reliability for the dmft/DMFT (ICC 0.88), PSI (Cronbach Alpha 0.57–0.77), and the recording of fillings/root canal treatments (ICC 3-surface filling 0.75; 4-surface filling 0.74; RCT 1.0). A weaker match was initially only found for diagnosing the need for filling replacement (ICC 0.41) which led to a retraining and discussion of achieving consensus on how to record a consensus on the teeth with different recordings. Dental trauma, hypoplasia, or malformations were excluded.

The pufa/PUFA index [12] was used to assess the clinical consequences of untreated caries and to estimate the treatment needs. Its components consist of pulp involvement, ulceration, fistula, and abscess in the primary or permanent dentition. The refugees were asked if they had any pain at the time of the examination or if they have had pain (sporadic or continues) any time before that. They were also asked if they had any emergency treatment due to acute dental pain. Palpation, mobility, and pain on percussion of questionable teeth were performed in order to diagnose pulpal involvements in combinations with the anamnestic reports by the refugees. Deep caries lesion with clear signs of the abovementioned parameters, fistula, or abscess formation was recorded as pulpal involvement. Refugees with acute pain, inflammation or abscess were sent to a dentist to have an emergency treatment to be relieved from their pain. Lesions in the surrounding tissues that are not associated with a tooth with visible pulp involvement as a result of caries were not recorded.

The findings were directly coded into an Excel database sheet (© Microsoft Office 2010, Version: 14.0), analyzed for the different age groups in Statistical Package for the Social Sciences software (© SPSS Inc., Version: 17.0) and compared with the representative cohorts in the German national surveys (3-, 6–7-, 12-, 35–44-, and 65–74-year-olds).

## Results

The 544 refugees (male: *n* = 339, 62.3%; female *n* = 205, 37.7%) were well distributed among the various age groups from 3 to 75 years (Table 1). The refugees came mainly from Syria, Afghanistan, and Iraq (*n* = 129; 23.7%; *n* = 92; 16.9% and *n* = 76; 13.9%, respectively). Other nationalities were present at lower percentages, mainly from Arabian countries (Egypt, Mauritania, Lebanon, Palestine, Morocco), Eastern Europe (Kosovo, Albania, Ukraine, Armenia, Serbia, Cheek Republic, Georgia, and Macedonia), and from Asia (Iran, Pakistan, Thailand, Azerbaijan, Tajikistan, and Russia) as well as from African countries (Eretria, Ghana, Nigeria, Ethiopia, and Somalia). This distribution mirrors the national German statistics on refugees arriving in Germany [3].

**Table 1:** Caries experience (dmft/DMFT) and its single components for the different age groups in refugees and in comparison with the German resident population [10, 11]

Age group (years)	*n*	*d*	*m*	*f*	dmft	*D*	*M*	*F*	DMFT	German residents dmft/DMFT
3	37	2.54 ± 3.6	0.05 ± 0.3	0.03 ± 0.2	2.62 ± 3.6	-	-	-	-	0.48 dmft (DAJ 2017)
6–7	73	4.21 ± 3.4	0.47 ± 1.1	0.55 ± 1.0	5.22 ± 3.4	0.12 ± 0.4	0.00 ± 0.0	0.02 ± 0.1	0.13 ± 0.5	1.73 dmft (DAJ 2017)
8–11	89	2.50 ± 2.4	0.53 ± 1.2	0.57 ± 1.1	3.60 ± 2.7	0.42 ± 0.9	0.02 ± 0.2	0.26 ± 0.8	0.70 ± 1.3	-
12	12	0.62 ± 0.8	0.08 ± 0.3	0.15 ± 0.6	0.85 ± 0.9	1.12 ± 1.3	0.06 ± 0.2	0.82 ± 1.6	2.00 ± 1.9	0.44 DMFT (DAJ 2017)
13–17	40					1.93 ± 2.0	0.23 ± 0.5	0.72 ± 1.4	2.87 ± 2.7	-
18–34	123					3.72 ± 3.0	1.46 ± 2.1	2.24 ± 3.8	7.43 ± 5.7	-
35–44	87					3.13 ± 3.0	3.22 ± 4.6	4.21 ± 4.6	10.55 ± 7.1	11.2 DMFT (IDZ 2016)
45–64	73					3.64 ± 4.1	7.63 ± 7.3	3.64 ± 4.3	14.92 ± 7.7	-

### Primary dentition

The caries experience in 3-year-olds was high (2.62 ± 3.6 dmft), mainly composed of open cavitation (2.54 ± 3.6 dt) and very little fillings or extractions (Table 1). The German reference group exhibits only one fifth of this caries level with a mean of 0.48 dmft, and only around 14% of the 3-year-olds in Germany did not have a naturally healthy dentition (dmft > 0, refugees 54%) [11]. Fifty-one percent of the 3-year-old refugees needed treatment (dt > 0), and 16% also showed a pufa index greater zero indicating caries complications (mean pufa: 2.33 ± 1.9 in these children, 0.38 ± 1.1 in all refugee children, Table 2).

**Table 2:** Caries prevalence (dt/DT & dmft/DMFT) and caries consequences (pufa/PUFA) in refugees according to age

Age group (years)	*n*	dt/DT = 0 *n* (%)	dmft/DMFT = 0 *n* (%)	pufa/PUFA = 0 *n* (%)	pufa/PUFA (Ø for all)	pufa/PUFA (Ø for > 0)
3	37	18 (49%)	17 (46%)	31 (84%)	0.38 ± 1.1	2.33 ± 1.9
6–7	73	10 (14%)	5 (7%)	45 (62%)	0.86 ± 1.4	2.25 ± 1.4
8–11	89	12 (14%)	12 (14%)	57 (64%)	0.80 ± 1.3	2.38 ± 1.3
12	12	2 (12%)	2 (12%)	14 (82%)	0.18 ± 0.4	1.00 ± 0.0
13–17	40	11 (28%)	9 (23%)	35 (88%)	0.18 ± 0.6	1.40 ± 0.9
18–34	123	12 (10%)	4 (3%)	92 (75%)	0.40 ± 0.8	1.58 ± 0.9
35–44	87	14 (16%)	4 (5%)	67 (77%)	0.37 ± 0.9	1.60 ± 1.2
45–64	73	15 (21%)	2 (3%)	50 (69%)	0.45 ± 0.8	1.43 ± 0.8

The highest caries prevalence in the primary dentition was found in 6–7-year-olds (5.22 ± 3.4 dmft), also mostly as untreated cavitation (4.21 ± 3.4 dt) and a few restorations (0.55 ± 1.0 ft) or extractions (0.47 ± 1.1 mt), and only 14% of the children in this age group were not affected by any visible caries defects at all (dt/DT = 0). Children residing in Germany have been presenting much lower caries levels for the last decades and a mean of 1.73 dmft in 2016 [10] (Table 1).

Only 7% of the 6–7-year-old refugees had a healthy primary dentition (dmft/DMFT = 0), in contrast to 56.4% [10] of German 6-7-year-old first graders. This was accompanied by fistulas, abscesses, etc. in 38% of the refugee children (pufa/PUFA > 0, mean 2.25 ± 1.4; 0.86 ± 1.4 in all refugee children, Table 2).

### Mixed dentition

For the 8–11-year-old refugee children, the caries levels decreased to 3.60 ± 2.7 dmft, also mostly untreated (2.50 ± 2.4 dt, Table 1). Likewise, in the permanent dentition, caries developed quickly (0.70 ± 1.3 DMFT) with open cavitation as the main component (0.42 ± 0.9 DT). Only 16 out of 89 children (18%) in this age group were caries-free (dt/DT = 0), and fewer children (*n* = 12, 14%) had mainly healthy dentitions (dmft/DMFT = 0). The high caries levels led to 36% of the refugees showing signs of pulp necrosis and subsequent consequences (pufa/PUFA > 0: mean 2.38 ± 1.3; vs. 0.80 ± 1.3 in all refugee children, Table 2).

### Adolescents

The WHO reference group of 12-year-olds [4] had a four times higher caries experience (mean 2.00 ± 1.9 DMFT, Table 1) and open cavitation (1.12 ± 1.3 DT) than their German counterparts (0.44 DMFT, 0.14 DT, resp., Table 1). Only 12% of the refugees were caries-free (dt/DT = 0) compared with 81.3% of the German residents [11]. The pufa/PUFA index was comparatively low (18% affected, mean 1.00 ± 0.0; 0.18 ± 0.4 in all refugee children, Table 2).

In the age group of 13–17-year-olds (*n* = 40), the DMFT mean value increased to reach (2.87 ± 2.7). The DT single component made the bulk amount from the total DMFT index value (DT = 1.93 ± 2.01). This age group was one of the subgroups that could not be compared with the German residents’ cohorts because it was not included in the recent DAJ survey [11]. In this age group specifically, 28% (*n* = 11) of the participants were caries-free (DT = 0), and almost a quarter of the participant in this age group (*n* = 9, 23%) had a dentition which was naturally healthy (dmft/DMFT = 0). The percentage without any subsequent complications (PUFA = 0) in the age group 13–17-year-olds was 88%, which was the highest percentage in this study (Table 2). The 12% of this age group with a positive PUFA index had an average value of 1.40 ± 0.9 (0.18 ± 0.6 mean PUFA in all refugee children).

### Adults

The largest group of refugees were young men from 18 to 34 years of age with moderate caries levels of 7.43 ± 5.7 DMFT which was often untreated (DT = 3.72 ± 3.0, Table 1 ), and 25% of them showed complications (mean PUFA 1.58 ± 0.9, Table 2).

There was a continuous increase in the number of decayed, filled, and missing teeth, ages 35 to 44 years with a mean DMFT of 10.55 ± 7.1. The German resident population has a slightly higher caries experience (11.2 DMFT) [11], but in contrast to the refugees, their treatment need is very low. There was a mean of only 0.5 carious defects in the German residents in contrast with 3.13 (± 3.0) decayed teeth in refugees, restorations, and extractions that had almost an equal number of 4.21 ± 4.6 FT and 3.22 ± 4.6 MT, but the German residents have fewer missing teeth (2.1 DMFT) [11], more restorations (8.6) [11], and lower DT component (0.5) compared with the refugees in the same age group when it comes to the MT and DT component values, 3.22 and 3.13, respectively (Table 1). The PUFA index had a maximum for adults of this age group with 23% being affected (mean 1.60 ± 1.2, Table 2).

The 45–64-year-old refugees had the highest total DMFT index (14.92 ± 7.7) with missing teeth accounting for the major part of it (7.63 ± 7.3 MT). The FT and DT components in this age group of refugees were almost similar, 3.64 ± 4.3 FT and 3.64 ± 4.1 DT, respectively (Table 1). A comparison with the German residents in this age group could not be performed due to the lack of data in the DMS study since this age group was not included [11]. Due to the chronification of many processes, caries consequences were found considerably less frequently than in children (mean PUFA = 0.45 ± 0.8). The mean PUFA index for 31% of the participants in this age group with a positive index value was 1.43 ± 0.8, while the other 69% of this age group showed no signs of complication (PUFA = 0) due to caries (Table 2).

## Discussion

Refugees and the consequences of their arrival in the host countries are a sensitive issue ethically, economically, and, of course, medically [8]. Due to their low socioeconomic status, the difficulty of the situation of their home countries being active war zones, the life threatening situations, and difficulties of traveling to the hosting countries, the refugees have several acute and chronic infections and diseases [6, 13].

To our knowledge, this was the first representative cross-sectional study on oral health in refugees. The largest sample of refugees collected in Germany (*n* = 544) for a study on oral health is also the first to cover the entire life span (3–74+ years). This is especially remarkable as one has to consider that mostly young men between 18 and 34 years of age flee from their home countries first and live in the initial welcome centers for the refugees until they get the right to stay in Germany. Then, they apply for family reunion, meaning that their children and the rest of the family members receive the right to follow as well which increases the need for gaining data on the other groups as well. To achieve valid samples of the subgroups of children, adolescents, and older refugees, over-recruitment of these groups was necessary. Due to the great responsiveness of the refugees to take part in the survey and the ability of the examiners to address most refugees in their Arabian native language, a selection bias could be reduced, and over-recruitment of the smaller refugee age groups allows for a representative few on the overall refugee population.

### Comparison with German population

The majority of the refugee children exhibited untreated caries in the primary dentition which led to a relevant proportion of fistulas, abscesses, and ulcerations after pulp necrosis. This means that every third child had unmet dental treatment needs. This was already true for 3-year-olds and increased up to 6–7 years of age. Similar values were found for German children 30 years ago, but the preventive system led to clear caries reductions [10, 11]. This is most pronounced in the age group of 12-year-olds, where 80% in Germany exhibit no past caries experience on a defect level, while this is true for only 12% of the refugees. Thus, the German health care system has achieved enormous health benefits in the dental field which should also be made available to the refugee children.

German adults who were born before the introduction of preventive services in the German health care system past the 1980s have presented with similar or even higher caries levels in childhood or adolescents than the refugees [10, 11] which explains while the dental health gap between German residents and refugees disappears for the adults.

### Comparison with other German studies

The few other studies performed on oral health in refugees in Germany did not have national wide recruitment and sometimes concentrate on Arabian refugees only, and they are limited to the selected age groups which usually exclude children, adolescents, or seniors [13, 14]. Due to the lower numbers, an age-specific subanalysis was not possible leading to a lower mean caries experience in all 16- or 18–64-year-olds than in our study (6.89 and 6.38 versus 10.96 DMFT), possibly due to the over-recruitment of older adults in the present study. The mean number of carious defects was similar (4.00 and 2.90 versus 3.49 DT), stressing the high unmet treatment needs in all refugees, which is also stressed by 11–49% of the refugees with immediate treatment needs due to pain or infection. Other subsequent problems of caries progression such as fistulas and abscesses (PUFA) were not recorded in these studies, although caries problems remain the most common dental disease in humans [15].

Interestingly, migration background is not always the main explanatory factor for higher caries levels in immigrant, as Splieth et al. [16] could show that after an adjustment for the socioeconomic background, the higher caries levels in migrants almost disappeared.

### Comparison with surveys worldwide

Only few and limited European studies regarding recent refugees concentrate on the main group of young male from the Middle East and Africa or children [17, 18], but they also find similar mean caries levels of 5 dmft in children or 7–8 DMFT in young adults, with also a high rate of untreated caries defects or even acute pain (38%). An exception is a Belgian study with 4037 refugees from all age groups examining primarily general medical problems [19]. Toothache represented the third most relevant concern with 10%, after cough and sore throat with 16% and 15%, respectively. Also, in a study on Lesbos/Greece which was one of the entrance gates for refugees to Europe, 28% of the children and adolescents had dental problems being the second most commonly diagnosed health problem after the respiratory infections [6]. This clearly shows that dental care is a very common and neglected issue in refugees caused by dietary problems, poor access to oral hygiene facilities, and oral health services [20]. Refugees also bring the high caries levels from their home countries without sufficient emphasis on prevention in their national health systems as in Syria or Iraq [21-24]. As the according DMFT values in adolescents match quite well with the data of this study, a high representativity can be assumed.

## Perspectives

As the high caries rate and its frequent complications in the recently arrived refugees in Germany presents a relevant problem, the issue of treatment and, most of all, prevention should be addressed on a national level. As the numbers of new refugees have decreased significantly, the impact on the German dental care delivery system will be a temporary phenomenon, but especially in the children and the primary dentition, the prevention gap between refugees and the German residents is most pronounced. Transferring the success of preventive dentistry in Europe to the refugees might eventually reduce the future treatment needs and costs. Special oral health prevention programs for refugees may be necessary to be implemented additionally, since the refugees comprise a collection of high-risk factors including the low economic level, low education level, language barrier, and the daily stress of the new lifestyle they live in [6, 13]. Thus, dentists visiting the refugees in their first arrival in the centers could help the refugees to take care to be more educated and aware of the importance of a healthy oral cavity.

As the results show, there are high rates of caries and a visible gap in prevention between the refugees coming to Germany and the German resident population. The free access to the existing preventive services in the German national health system for the refugees is granted after their acceptance which will help them to improve their oral health and to reduce the costs for future treatment. It would be important to integrate additional information for the refugees into the language and integration courses regarding dental health and dental prevention, since many refugees had untreated caries lesions.
